# Is value-based healthcare a strategy to achieve universal health coverage that includes oral health? An Australian case study

**DOI:** 10.1057/s41271-023-00414-9

**Published:** 2023-05-04

**Authors:** Tan M. Nguyen, Gemma Bridge, Martin Hall, Katy Theodore, Clare Lin, Ben Scully, Ruth Heredia, Long K.-D Le, Cathrine Mihalopoulos, Hanny Calache

**Affiliations:** 1 Deakin Health Economics, Deakin University, Level 3, Building BC, 221 Burwood Highway, Burwood, Melbourne, VIC 3125 Australia; 2 Dental Health Services Victoria, Level 1, Corporate Services, 720 Swanston Street, Carlton, Melbourne, VIC 3053 Australia; 3 Public Health and Preventive Medicine, Monash University, Level 4, 553 St Kilda Road, Melbourne, VIC 3004 Australia; 4 Queen Mary University of London, Mile End Road, London, E1 4NS UK; 5 Melbourne School of Population and Global Health, The University of Melbourne, Parkville, VIC 3010 Australia; 6 Melbourne Dental School, The University of Melbourne, Parkville, VIC 3010 Australia; 7 La Trobe University, Bendigo, VIC 3552 Australia

**Keywords:** Universal health care, Primary health care, Health policy, Allocation of health-care resources, Oral health, Noncommunicable diseases

## Abstract

The 2021 Resolution on Oral Health by the 74th World Health Assembly supports an important health policy direction: inclusion of oral health in universal health coverage. Many healthcare systems worldwide have not yet addressed oral diseases effectively. The adoption of value-based healthcare (VBHC) reorients health services towards outcomes. Evidence indicates that VBHC initiatives are improving health outcomes, client experiences of healthcare, and reducing costs to healthcare systems. No comprehensive VBHC approach has been applied to the oral health context. Dental Health Services Victoria (DHSV), an Australian state government entity, commenced a VBHC agenda in 2016 and is continuing its efforts in oral healthcare reform. This paper explores a VBHC case study showing promise for achieving universal health coverage that includes oral health. DHSV applied the VBHC due to its flexibility in scope, consideration of a health workforce with a mix of skills, and alternative funding models other than fee-for-service.

## Key messages

- Globally, universal health coverage has largely excluded oral health, despite its importance to overall health and wellbeing.
- The value-based healthcare agenda offers potential to support inclusion of oral health within universal health coverage in a cost-effective way.
- A comprehensive approach to oral healthcare reform can guided by applying the value-based healthcare agenda.

## Introduction

Achieving universal health coverage is critical for advancing the United Nations Sustainable Development Goal 3 to “Ensure healthy lives and promote well-being for all at all ages” [1]. At the end of 2021, almost half the world’s population lacked essential health services [2]. The World Health Organization defines universal health coverage as the ability for all citizens “access to quality health services they need without financial hardship”, which “encompasses health promotion and prevention, treatment, rehabilitation and palliative care across the life course” [3].

Preventing oral diseases is a major public health challenge that affects over 3.5 billion people globally [4]. The three most common oral diseases are dental caries, periodontal diseases, and oral cancer [4–6]. Oral health incorporates physical, psychological, emotional, and social domains that are integral to overall health and wellbeing [5]. A functional dentition enables adequate chewing ability and nutrition, and good oral health supports socialization. Many healthcare systems have not successfully tackled the global burden of oral diseases [4–6]. Dental services are often left to the private sector and governments place low priority on public dental services [7, 8].

The landmark ‘Resolution on Oral Health’ by the 74th World Health Assembly in 2021, which acknowledged that oral health should be a part of universal health coverage [9], signifies an important tipping point for oral health policy worldwide. In this viewpoint, justification is provided to integrate oral health into universal health coverage, its importance to overall general health and wellbeing, and explores the variety of health providers offering oral healthcare globally. An Australian case study is presented to illustrate how universal health coverage that includes oral health may become more achievable with adoption of a value-based healthcare (VBHC) agenda.

## Oral diseases and implications for overall health and wellbeing

Several systematic reviews have shown associations between oral diseases and non-communicable diseases, including cardiovascular diseases [10], depression [11], dementia and cognitive impairment [12, 13], diabetes mellitus [14], and obesity [15, 16]. In addition, oral diseases and increasing levels of tooth loss directly affect facial aesthetics, speech, oral function, social inclusion [17], and employment opportunities [18].

As with other non-communicable diseases, the prevalence of oral diseases follows a social gradient [19, 20], whereby oral health and quality of life worsen as socioeconomic disadvantage increases. Longitudinal studies corroborate this observation by demonstrating that greater socioeconomic disadvantage is associated with greater prevalence of dental caries, worse oral health (such as periodontal diseases, severe tooth loss and edentulism), and lower utilization of dental services [21–25]. The social determinants have a substantial impact on health [26–28], including oral health outcomes.

Common oral diseases share common and modifiable risks factors with other non-communicable diseases. Such risk factors include sugar consumption, tobacco use, and harmful alcohol consumption [4–6, 29]. The focus for preventing oral diseases has, for historical reasons, remained separate from mainstream medicine [6]. This fragmentation inhibits effective coordination of resources and efforts to reduce the oral disease burden [6] and its potential impact on other non-communicable diseases.

The direct cost of oral diseases accounts for 4.8% of global health expenditure (US$387 billion) annually, with an additional US$323 billion attributable to ‘indirect costs’, defined as productivity losses due to untreated caries, severe periodontitis, and severe tooth loss [30]. In Europe, €90 billion in direct costs for management of oral diseases ranks as the continents’ third most costly health expenditure, after costs for management of diabetes and heart disease at €119 billion and €111 billion, respectively [31]. Despite the high costs to society, oral healthcare expenditure is a fraction of total health expenditure. Estimates from 23 European countries show that the current dental expenditure as a fraction of the total health expenditure, in 2019, ranged from 2.5% in the United Kingdom to 9.6% in Estonia [30].

## Summary of oral healthcare systems

Globally, the administration and financing of oral health has remained external to healthcare systems. In Western countries such as the United States, Canada, Australia, and New Zealand, governments provide limited public dental services provided by government employees and leave provision and payment of most oral healthcare to the private sector, either subsidized through private health insurance or funded by the individual as an out-of-pocket expense [7, 8, 32, 33].

In Europe there is an increasing public policy appetite towards privatization [34, 35]. Publicly funded dental services provided for children by salaried dental practitioners are common in many European countries. This contrasts with services for adults for which widespread reliance on private dental care subsidized by the government persists [34]. Policies for the populations eligible for public dental services vary, as does utilization of the non-dentist oral health workforce (such as oral health therapists), and the type of dental services provided. In contrast, Scandinavian countries are well-known for having established universal health coverage that includes oral healthcare provided by government entities [34].

In other parts of the world, there are documented examples of universal health coverage that include oral health, in Brazil [36], Japan [37], South Korea [38], Taiwan [39], and Thailand [40]. The type of dental services funded by government varies, but countries that have introduced them more recently have used an incremental approach. Examples include expanding government funding for fissure sealants in South Korea [38, 41] and scaling and non-surgical periodontal treatment in South Korea [42–45] and Taiwan [46]. There is limited peer-reviewed documentation from low-income countries on government funding for oral healthcare across all age groups compared to middle-income and high-income countries [47, 48].

Primary oral healthcare is more common in high-income countries such as the Scandinavian countries and least common in Sub-Saharan African countries [49]. Currently, prevention and management of oral diseases have remained the assumed traditional role of the dental profession [6]. Importantly, countries that have more redistributive and universal welfare policies, including universal health coverage for oral healthcare, tend to have better population oral health [50–52].

## Global perspectives for the value-based healthcare agenda

Healthcare systems worldwide are constrained by budget restrictions and are increasingly challenged by population growth, complex healthcare needs, and changing client expectations. The trend towards paying for outcomes, known as VBHC, received particular attention when Porter and Teisberg articulated how healthcare should be delivered and measured [53]. The first driver for transforming healthcare systems is to define value. Porter and Teisberg define value by the health outcomes relevant to the clients divided by the costs for delivering those health outcomes [53, 54]. Increasing value occurs when analysts identify cost-efficiencies by minimizing appropriate costs to achieve the same outcome or achieving improved outcomes at the same cost.

Underpinning VBHC are six pillars that are interdependent and mutually reinforcing [53, 54]:

1. Organize into Integrated Practice Units,
2. Measure outcomes and costs for every client,
3. Move to bundled payment for care cycles,
4. Integrate care delivery across separate facilities,
5. Expand excellent services across geography, and
6. Build an enabling information technology platform.

These VBHC components are not new but bringing them together in a systematic approach to improving health outcomes has attracted attention from health funding bodies. A 2021 systematic review of VBHC initiatives in practice showed this approach was meeting its objectives including cost-savings, improving clinical and client-reported outcomes, and improving healthcare system efficiencies [55].

The literature reports limited VBHC initiatives for oral health. Jivraj et al. reported four case studies: developing Integrated Practice Units, implementing client-reported outcomes, cost accounting, and determining bundled payments, which remunerates for an episode of care, rather than a fee-for-service funding model, which renumerates each service or activity [56]. Riley et al. have also proposed several options for value-based payments [57] to move towards bundled payments for full care cycles.

The 2021 systematic review by Conquest et al. on bundled payments and fee-for-service payment models reported that clients favored bundled payments, although moving from fee-for-service to capitation could reduce services provided, including preventive interventions [58]. This review also suggested that adjusting the way dental practitioners are remunerated is unlikely to achieve all policy goals [58]; potentially elusive ones address oral health inequities and creating incentives for optimizing the dental workforce skill mix [58].

There are a range of dental practitioners who provide dental services (Appendix: Glossary). In Australia, oral health therapists are registered dental practitioners, who can provide diagnostic, preventive and dental treatment services alongside dentists. The Australian national oral health therapist to dentist dental workforce ratio is 1:4, compared to the Victorian public dental workforce ratio of 2:3 [59]. The difference has meant it is costlier to provide public dental services, nationally. Australia has not operationalized provision of oral healthcare for efficiency, partly due to an imbalance in the dental workforce skill mix [59]. There is also evidence that Australia lacks a strong prevention focus on public oral healthcare [60]. Prevention efforts can be increased and improved by establishing a dental service performance audit and feedback system and supporting appropriate continuing professional development for dental practitioners [61].

Because of these issues and complexities, to date, there have been few attempts to implement comprehensively all six pillars of VBHC internationally [62]. The continued focus of governments on increasing access to public dental services has demonstrated that achieving universal health coverage that includes oral health will not address population oral health needs sufficiently. VBHC can do this by being flexible in how care is delivered through a preventive and person-centered approach to care.

## Implementing value-based healthcare for oral health: an Australian case study

Dental Health Services Victoria (DHSV) is the leading public dental agency in the state of Victoria, Australia. It commissions 50 separate community health service entities to provide public dental services, and is itself a service provider, primarily for emergencies, clients with complex medical conditions, and dental specialist services. All Australian publicly funded state and territory public dental services vary in their administration and are heavily subsidized for low-income clients.

**Fig. 1:**
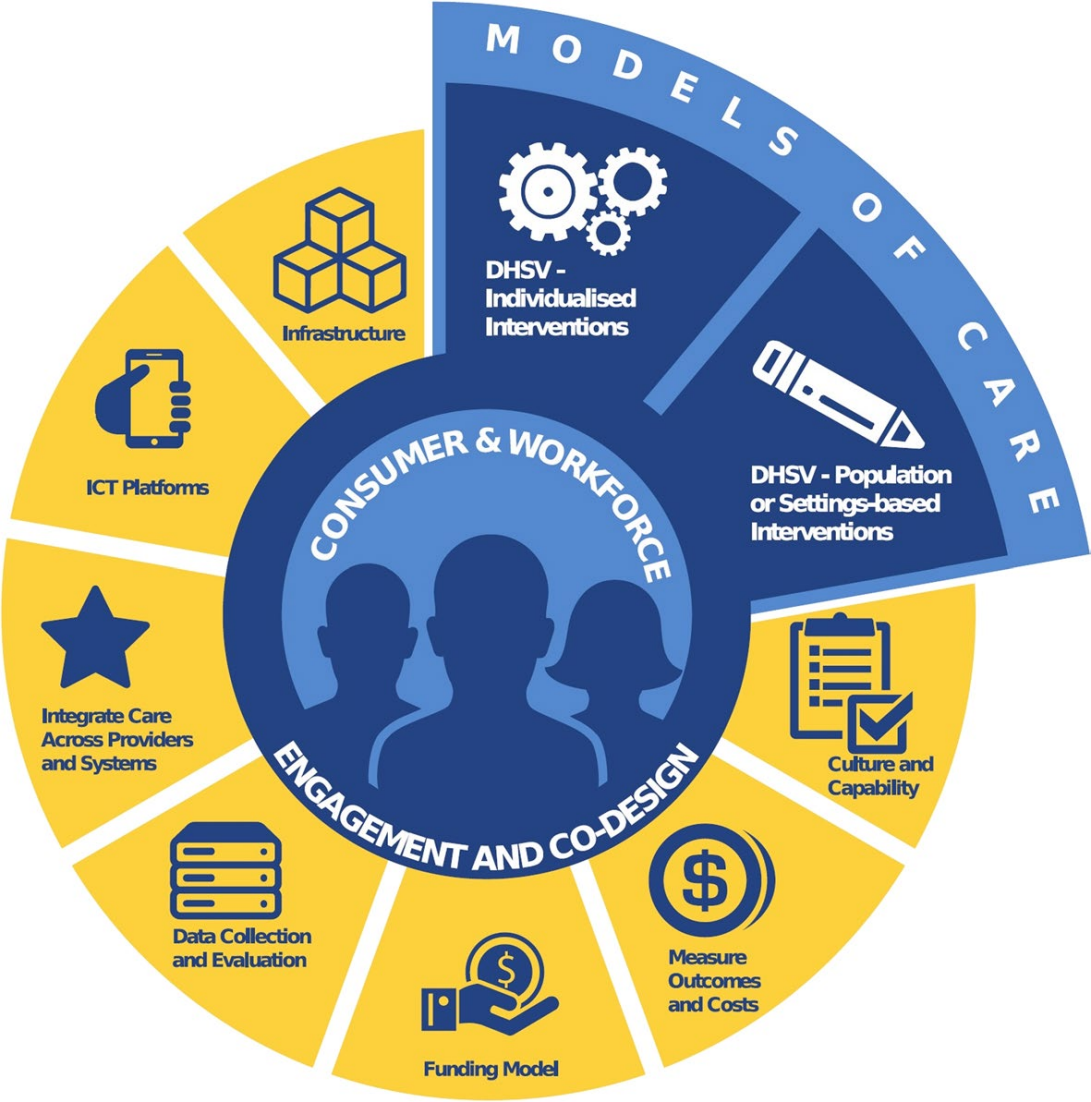
Dental Health Services Victoria conceptual framework using the value-based healthcare approach to service co-design

In 2016, DHSV began to transform provision of oral healthcare in the public dental services by exploring the application of VBHC to the oral health context [63] (See Fig. 1 for DHSV’s conceptual framework). Primarily, DHSV wanted to maximize use of the then current funding resources, which provided care to only 25% of the eligible population in Victorian public dental services, annually [64].

The VBHC agenda was not new to the Victorian Department of Health. The evolution and institutionalization of VBHC in Australia began in 1988, with the centralization of public healthcare funding procurement under the Health Services Act (1988) [65]. Government reviews of public healthcare funding procurement from 2011 to 2015 increased visibility and accountability for shifting the approach from short-term costs to long-term value and outcomes [65].

The Victorian Auditor General’s Office conducted a review of access to Victorian public dental services in 2016 [64], and a second, a follow-up, in 2019 [63]. During this time, the Australian federal government’s Productivity Commission provided a review to improve public dental services in all Australian jurisdictions [66]. The transition away from fee-for-service funding models and towards funding for outcomes also led to investments in information technology infrastructure to digitalize operations for moving toward a client-centric and digital health-care system [65].

Australian dental services, as is true for other areas of healthcare, are provided by a broad range of registered dental practitioners and dental support staff. Because dental caries and periodontal diseases are chronic non-communicable diseases, they are influenced by the social determinants of health [67] and are amendable to upstream preventive interventions as well as activities that support behavior change and self-management [68, 69]. Yet, the current funding model in Victoria, being fee-for-service, is one that rewards output (including time-consuming and expensive surgical interventions) and utilizes a highly skilled dental workforce, made up of dentists to provide dental services [64, 66].

Building workforce capacity is important to enable VBHC. Means to do so include enhancing the skills of dental assistants to provide oral health education and regular application of fluoride varnish [63], a medicament that has demonstrated strong effectiveness [70] and cost-effectiveness [71, 72] in preventing dental caries. DHSV committed to a key concept: to provide the ‘right care’ to the ‘right person’, at the ‘right time’ by the ‘right clinician’ at the 'right' place (dental practitioners and dental assistants with advanced training to work the full scope of practice).

DHSV is developing standard care pathways based on oral disease risk. It aims to provide high value, preventive-focused and evidence informed oral healthcare, and to minimize wait times. DHSV designed these pathways to maximize the reach of oral healthcare services and achieve better oral health outcomes for clients, key elements for achieving universal health coverage that includes oral health.

VBHC is a new concept for developing and implementing oral healthcare reform. To determine value, government entities and service provider need to establish a standard set of measurement outcomes for oral health that are useful for tracking progress in population health and ensuring the provision of oral healthcare is improving people’s lives. The purpose of having relevant oral health outcomes is to inform: (1) dental care improvement, (2) medical-dental integration, (3) value-based payments, (4) public health programs, and (5) monitoring and needs-based planning [73]. List1 described seven oral health measurement items [73]. Since then, a cross-national collaboration through the International Consortium for Health Outcomes Measurement, which included DHSV as a member, agreed on 31 conceptual outcomes included in the Adult Oral Health Standard Set [74].

DHSV used the standard set to develop a Victorian-specific Oral Health Questionnaire to understand the clients’ general and oral health needs, which was co-designed with consumers. Clients completed it using an online platform or by phone call. To identify clients with higher oral disease risk and oral health needs to prioritize clients for care in a timely manner, the Oral Health Questionnaire contains an algorithm with variable weighting to calculate a client’s oral disease risk and to rate oral health needs.

The algorithm is subject to validation and revision based on the continuous learning, feedback and data collected at DHSV. It is possible that VBHC may create perverse incentives whereby health professionals may select clients more likely to have ‘better’ health outcomes. DHSV developed the Oral Health Questionnaire to prioritize clients with greater oral health needs to access timelier public dental services. Details of the Oral Health Questionnaire and its evaluation is planned by DHSV in a forthcoming peer-reviewed publication.

## Dental Health Services Victoria Adult Service Delivery Model

DHSV has been ambitious. One priority is to develop the Adult Service Delivery Model to address the current fragmented delivery of public dental services. Internal reviews show clinical variation across oral healthcare in Victoria. In addition, the model does not prioritize provision of services based on oral disease risk or oral health needs [66], which can exacerbate oral health inequities.

DHSV has completed ‘proof of concept’ in 2018–2019 to inform the development of an Adult Service Delivery Model. It found that clients involved in the ‘proof of concept’ received more preventive dental services and lower rates of clients failed to attend their dental appointments [63]. A sizable proportion of dental services could be performed by other dental team members, thereby freeing up the dentist with the widest scope of practice to manage more complex client health conditions [63].

Additionally, service providers are concerned regarding the potential lack of oral healthcare literacy and engagement of clients with oral health self-care management. To address this, group introductory education session for clients, which was included in DHSV’s ‘proof of concept’, provided by dental support staff provided information about the scope of public dental services, their rights and responsibilities, healthcare pathways and oral health education. An evaluation demonstrated that over 80% of clients attended a scheduled group introductory session appointment, with 95% having followed up with a dental appointment compared to standard care [63].

DHSV applied the six strategies articulated by Porter and Lee [54], which aim to achieve universal health coverage that includes oral health, by

1. Organizing integrated practice units: Integrated practice units involve clinical and non-clinical team members organized to coordinate care of the clients’ oral health needs. Guidance documents are being developed to support Victorian community health services in utilizing the skills of all dental team members to their full scope of clinical practice, and eventually, integrate medical-dental services where appropriate.
2. Measuring outcomes and costs for every client: using the Oral Health Questionnaire to inform measurement outcomes, DHSV can assess individual and population risk factors and patient-reported outcome measures by the end of 2023. Measurement is important to establish performance benchmarks to ensure minimal clinical variation to achieve good outcomes and redirect resources to expand public dental services to more of the eligible population.
3. Moving to bundled payment for care cycles—DHSV and the Victorian Department of Health have engaged consultants to establish a conceptual bundled payment funding model to pay for outcomes [63].
4. Integrating delivery of care across separate facilities in a region: organizing community health services to deliver the Adult Service Delivery Model, and wherever possible, provide specialist dental services locally. Non-dental practitioners would provide preventive services such as Aboriginal and Torres Strait Islander health practitioners in applying fluoride varnish for children. Although some community health services have merged out of necessity to pool resources and increase efficiency, DHSV has established regional clinical leadership to foster cooperation and coordination at the local level.
5. Expanding high quality services across geography: DHSV is establishing regional specialist dental services hubs with ‘satellite’ community health service sites by the end of 2023. This differs vastly from the only available option for eligible clients to have specialist dental services at the Royal Dental Hospital of Melbourne. This step requires clinical leadership and building capacity of the dental workforce, and with telehealth capability as part of routine practice. As the standard care pathways mature, integration of non-dental practitioners can add greater value to the VBHC agenda by working with nurses, midwives, general practitioners, pharmacists, and other allied health practitioners to provide preventive oral healthcare services and initiate referrals for oral healthcare.
6. Building an enabling information technology platform: development of a user-friendly and centralized information technology platform is an essential driving force in achieving the objectives of VBHC. DHSV and the Victorian Department of Health are working together to build information technology support for a state-wide electronic health record for public dental services, with integration with content management software to support client communication. For the Adult Service Delivery Model to succeed, it will be essential to leverage telehealth coaching capability to enable individualized oral health ‘check-ins’ by dental assistants with advanced training [75], also known as oral health educators [76]. Re-orientation of the oral health workforce is an essential first step to improve efficiency in service delivery [77]. Community health services will need support by a centralized information technology platform to enable tracking of cost and outcomes for continuing quality improvement.

## Limitations

DHSV’s adoption of VBHC is a promising step towards universal health care that includes oral health. The new approach can redirect use of limited resources for oral healthcare to deliver better care to a greater proportion of the population. Potential positive impacts in undertaking the DHSV’s VBHC agenda remain to be measured through robust evaluation. It will be important to identify any perverse incentives that might arise from a shift to VBHC. Key barriers to achieving the goals for the new model include insufficient funding commitment by governments to provide oral healthcare, and continuation of traditional fee-for-service payment models that reward outputs rather than outcomes. Increasing services to more people alone would not satisfy the need for oral health care among priority, and currently underserved populations. Universal health coverage that includes oral health should include strategies focused on prevention, reduce oral health inequities, and tailored to diverse needs of populations.

## Conclusion

The DHSV experience has highlighted that changing the healthcare system and entrenched ways of service delivery can be challenging given limited robust evidence for guidance, but simply ‘starting’ is critical. Operationalizing the ‘proof of concept’ elements of VBHC with staff and clients may take time, it provided insights for long-term oral healthcare reform. Although the objectives of DHSV’s VBHC agenda are not explicitly directed to achieve universal health coverage that includes oral health, this approach can expand population access to public dental services and financial protection as the next step towards it.

## Appendix: glossary

Dental practitioners are the term to described registered health practitioners who are providers of dental services in Australia. It includes:

Dental specialists—dentists with advanced training and/or qualifications who provide dental care in a particular dental specialty.

Dentists—dental practitioners who provide dental care within the broadest range of services in dentistry.

Dental prosthetists—dental practitioners who provide dental care via the assessment, fabrication, and management of a dental prosthesis.

Oral health therapists—dental practitioners who provide dental care in diagnostic, prevention and treatment of oral diseases. They have skillsets as a dental therapist and a dental hygienist.

Dental therapists—– dental practitioners who provide dental care in diagnostic, prevention and treatment of oral diseases, primarily focused on dental caries.

Dental hygienists—dental practitioners who provide dental care in diagnostic, prevention and treatment of oral diseases, primarily focused on periodontal diseases.
